# Thin Films of Chlorinated Vanadyl Phthalocyanines as Active Layers of Chemiresistive Sensors for the Detection of Ammonia

**DOI:** 10.3390/mi14091773

**Published:** 2023-09-15

**Authors:** Darya Klyamer, Alexandr Sukhikh, Dmitry Bonegardt, Pavel Krasnov, Pavel Popovetskiy, Tamara Basova

**Affiliations:** 1 Nikolaev Institute of Inorganic Chemistry SB RAS, 3 Lavrentiev Pr., 630090 Novosibirsk, Russia; klyamer@niic.nsc.ru (D.K.); a_sukhikh@niic.nsc.ru (A.S.); bonegardt@niic.nsc.ru (D.B.); popovetskiy@niic.nsc.ru (P.P.); 2 International Research Center of Spectroscopy and Quantum Chemistry, Siberian Federal University, 26 Kirensky St., 660074 Krasnoyarsk, Russia; kpo1980@gmail.com

**Keywords:** vanadyl phthalocyanines, chemiresistive sensors, gas sensors, ammonia

## Abstract

Halogenated metal phthalocyanines are promising materials for the manufacture of active layers of chemiresistive sensors for the detection of various gases. Despite the high interest in such sensors, there are few systematic studies of the position of halogen substituents in phthalocyanine macroring on the chemiresistive response of their films to gases. In this work, we prepared and studied films of novel tetrachlorosubstituted vanadyl phthalocyanine derivatives with Cl substituents in the peripheral (VOPcCl_4_-p) and nonperipheral (VOPcCl_4_-np) positions of the phthalocyanine ring as active layers of chemiresistive sensors to reveal the effect of the position of substituents on their structure and sensor response to low concentrations of NH_3_. It was shown that the films of VOPcCl_4_-p exhibited a noticeably higher sensor response to NH_3_ than the VOPcCl_4_-np ones. The limit of detection of NH_3_ was 0.7 ppm. The sensing layers demonstrated a reversible sensor response at room temperature with fairly low response/recovery times. It was also demonstrated that NH_3_ can be detected in the presence of various interfering gases (CO_2_ and H_2_) and some volatile organic vapors, as well as in a mixture of gases with a composition close to exhaled air.

## 1. Introduction

Ammonia is a colorless gas with a specific smell. The main consumers of ammonia are agricultural enterprises, although it is also used in various household and industrial cleaning products, as well as in industrial metalworking processes, the paper industry, and industrial refrigeration units [1]. Ammonia has a strong corrosive effect and causes severe irritation of the skin, eyes, mucous membranes of the mouth, and respiratory and digestive systems [2]. Its concentration above 100 ppm can cause burning in the eyes, lacrimation, swelling of the eyelids, corneal abrasion, blurred vision, and even blindness. The detection of ammonia is an important task not only in industry but also in medicine [3,4]. Ammonia is used as a biomarker for the diagnostics of disease by analyzing its content in breath air. For example, an increased level of NH_3_ in exhaled air of more than 2 ppm is a sign of renal pathologies [5,6].

Currently, electrochemical and luminescent sensors, as well as gas chromatography–mass spectrometry are most often used to analyze these gases in exhaled air [7,8,9]. The development of new semiconductor sensors for the analysis of gas biomarkers is of particular interest due to their cheapness, portability, and speed. Oxides [10,11], transition metal nitrides [12,13], nanocarbon-containing materials [14], and metal phthalocyanines [4,15,16] are widely used as active layers of chemiresistive sensors. The latter attract attention due to the possibility of widely varying their resistive and electrocatalytic properties by changing their molecular structure (e.g., central cations and substituents in the macroring or axial positions), as well as the morphology and orientation of their films. Another advantage of MPc layers is the reversibility of their response at room temperature without additional heating, as well as the possibility of creating sensing layers on flexible substrates [17,18,19].

Among the variety of metal phthalocyanines, phthalocyanines with various substituents in axial positions can be distinguished, for example, phthalocyanines of gallium and aluminum MXPc (M = Al, Ga; X = Cl, OH, F), as well as phthalocyanines of titanyl and vanadyl (TiOPc and VOPc). Such phthalocyanines have a packaging type different from phthalocyanines M (II) Pc (M = Mg, Cu, Co, Ni), as they have a flat structure and additional places of interaction with gas analytes [20,21]. For instance, unlike other 3D-metal phthalocyanines, VOPc and its substituted derivative, VOPcR_n_ (n = 4, 8, 16), are nonplanar molecules with vanadyl groups located perpendicular to the plane of aromatic rings [22,23]. This leads to two-dimensional π-π stacking in the crystal lattice and, as a consequence, to shorter intermolecular distances and greater overlap of the π-π stacking compared to planar phthalocyanines, which ultimately contributes to the higher mobility of charge carriers [24]. These properties open up the prospect of using them in highly productive organic thin-film field-effect transistors (OFETs) and sensors.

Ultrathin vanadyl phthalocyanine films were used as sensing layers of chemiresistors for the detection of nitrogen dioxide at room temperature [25]. Surface-type capacitive humidity sensors based on evaporated VOPc films were studied by two different groups of authors [26,27]. Ji, et al. [28] fabricated an ultra-thin highly ordered heterojunction-structured device based on multilayers of VOPc, N,N′-diphenyl perylene tetracarboxylic diimide (PTCDI-Ph), and para-hexa-phenyl (p-6P), which was very sensitive to NO_2_ and had a nearly five times larger response than the corresponding single-heterojunction device.

**Figure 1:**
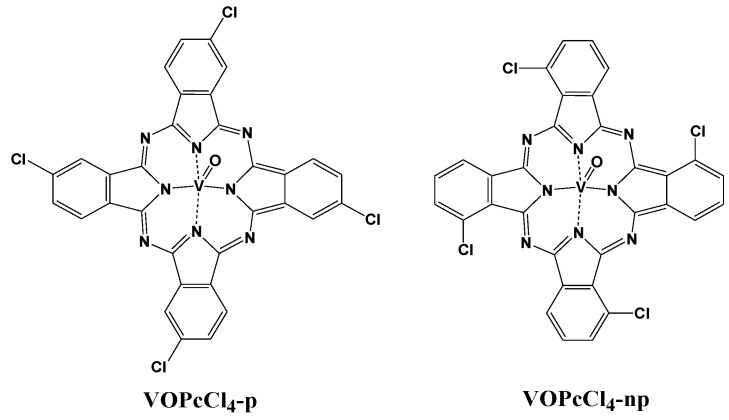
Vanadyl phthalocyanines with Cl substituents in the peripheral (VOPcCl_4_-p) and nonperipheral (VOPcCl_4_-np) positions of phthalocyanine macrocycle.

Many authors show in their works that the introduction of halogen substitutes into the phthalocyanine macrocycles results in improvements in their sensitivity to electron-donating gases [29,30]. In tetrasubstituted phthalocyanine molecules, halogen substituents can be introduced into both peripheral (MPcX_4_-p) and nonperipheral (MPcX_4_-np) positions of the phthalocyanine macroring (Figure 1). In our previous work, thin films of unsubstituted and tetrafluorosubstituted vanadyl phthalocyanine derivatives (VOPc and VOPcF_4_-p) were used for the detection of gaseous ammonia and hydrogen. It was shown that VOPcF_4_-p films demonstrated a 2–3 times higher sensor response to NH_3_ compared to VOPc films [31]. In turn, a comparative analysis of the sensor response to ammonia showed that the films of VOPcF_4_-p exhibited a greater sensor response than those of VOPcF_4_-np [32]. This result correlated well with DFT calculations, which revealed stronger binding of NH_3_ and VOPcF_4_-p molecules.

It was shown using the example of zinc phthalocyanines that films of chlorosubstituted metal phthalocyanines demonstrated an even higher chemiresistive sensor response to ammonia than fluorinated ones [30], but the structure and sensor properties of chloro-substituted oxometallo phthalocyanine films have not yet been studied.

In this work, we prepared and studied films of novel tetrachlorosubstituted vanadyl phthalocyanines with Cl substituents in the peripheral (VOPcCl_4_-p) and nonperipheral (VOPcCl_4_-np) positions of the phthalocyanine ring (Figure 1) as active layers for detecting low concentrations of NH_3_ and compared their sensor performance with their fluorinated analogs. The novelty of this work consists of expanding the set of metal phthalocyanines suitable for use as sensor layers and revealing the influence of the position of substituents in the macroring on the structure of films and the sensor response to the gaseous analytes. The sensor characteristics of the sensors (viz reversibility, limit of detection, response, and recovery times) were determined. The sensing layers were tested in the presence of various interfering gases (CO_2_ and H_2_) and some volatile organic vapors, as well as in a mixture of gases simulating exhaled air.

## 2. Materials and Methods

### 2.1. Preparation and Characterization of Thin Films

VOPcCl_4_-p and VOPcCl_4_-np were synthesized according to the standard template synthesis method by melting the mixture (1:4 molar ratio) of VOCl_3_ with corresponding 4-chlorophthalonitrile (abcr, CAS 17654-68-1) or 3-chlorophthalonitrile (abcr, CAS 76241-79-7). The compounds were purified by vacuum sublimation (10^−5^ Torr, 420–450 °C). Thin films of VOPcCl_4_-p and VOPcCl_4_-np were deposited by a physical vapor deposition method, using a vacuum universal station VUP-5M (Sumy, Ukraine). The residual pressure was 10^−5^ Torr, the evaporation temperature was 420–450 °C, and the substrate temperature was about 60 °C.

Thicknesses of the VOPcCl_4_-p and VOPcCl_4_-np films were 82 and 87 nm, respectively. The choice of film thickness was based on preliminary studies. It was previously shown that films of smaller thickness often do not form a continuous film on the surface of the electrodes, which prevents their good conductivity. Films with a thickness of more than 100 nm have low sensor sensitivity and a long response time due to the difficulty of gas diffusion into the film. The thickness of the films was determined through spectroscopic ellipsometry, using a spectroscopic ellipsometer ELLIPS 1771 SA (ISP, Novosibirsk, Russia) according to the previously described technique [33].

An XRD analysis of VOPcCl_4_-p and VOPcCl_4_-np single crystals was performed using A Bruker D8 Venture (Bruker AXS Inc., Billerica, MA, USA) single-crystal diffractometer (Photon III C14 CPAD detector, MoKα Incoatec IμS 3.0 microfocus X-ray source, 3-circle kappa-goniometer). The temperature of the crystals was 150 (1) K and was controlled using an open-flow nitrogen cooler (Cryostream 800 plus, Oxford Cryosystems, Oxford, UK). Several standard ω-scans with a frame width of 0.5° were used to collect the data. Data collection, reduction, absorption correction, and global unit cell refinement were performed in the APEX 3 [34] software package (V2018.7–2 (SAINT 8.38A, SADABS−2016/2), Madison, WI, USA). Reduced hklF datasets were then processed in Olex2 1.5 software [35] and with SHELXT v.2018/2 for the initial structure solution [36] and with SHELXL v.2018/3 [37] for the subsequent refinement. Finalized CIF files were deposited to The Cambridge Crystallographic Data Center (CCDC) with the numbers 2267526, 2267527, and 2267528 and can be obtained free of charge at www.ccdc.cam.ac.uk/structures, accessed on 5 June 2023.

An XRD study of thin films and bulk powders of VOPcCl_4_-p and VOPcCl_4_-np was performed using a Bruker D8 Advance powder diffractometer (Bruker AXS Inc., Billerica, MA, USA) in the Bragg–Brentano scheme with a vertical θ-θ goniometer (CuKα sealed tube, 0.01° scan step).

AFM images of the films were obtained using the Ntegra Prima II nanolaboratory (NT-MDT, Moscow, Russia). The HA_NC probe parameters were described in our previous work [30]. A scanning electron microscope (JEOL–JSM 6700 F, Tokyo, Japan) was used for the characterization of the films’ surface morphology.

Optical absorption spectra were recorded with (OKB SPECTR LLC, Saint-Petersburg, Russia), while Raman spectra were obtained using a LabRAM Horiba single spectrometer (Montpellier, France) (488 nm line of an Ar+ laser).

### 2.2. Measurements of the Sensor Response

The chemiresistive response of VOPcCl_4_-p and VOPcCl_4_-np films was investigated by measuring the change in resistance upon the introduction of ammonia in the concentration range of 1–50 ppm. The resistance was measured at the constant voltage of 10 V, using an electrometer Keithley 236 (Tektronix Inc., Beaverton, OR, USA). To measure the sensor response, ammonia of the required concentration and air used for purging were alternately injected into the flow cell. Most of the experiments were conducted in dynamic mode. In this mode, ammonia was injected at a constant flow rate of 300 mL/min. The exposure time was 15 s. Static mode was used only to determine the response and recovery times of the sensors. In static mode, the airflow was passed through the chamber until the film resistance reached a constant value, and then the chamber valves were closed and NH_3_ of the required concentration was injected into the cell. The resistance was recorded after the saturation current was reached. After that, the chamber was opened and blown by a stream of air. All measurements were carried out at room temperature (22 ± 2 °C).

The sensor response values were calculated: (R − R_o_)/R_o_. (R is the steady resistance of the film at a certain analyte concentration, and R_o_ is the initial resistance of the film in air). The measurements of the response of three deposited films were used to calculate the standard deviation. The source of NH_3_ was a tank with pure commercial (“Chistye Gasy+”, Novosibirsk, Russia) gas (1%) diluted in dry argon. The required gas concentrations were set using the mass flow controllers. Dry air was used as a diluting and purging gas.

### 2.3. Theoretical Calculations

**Figure 2:**
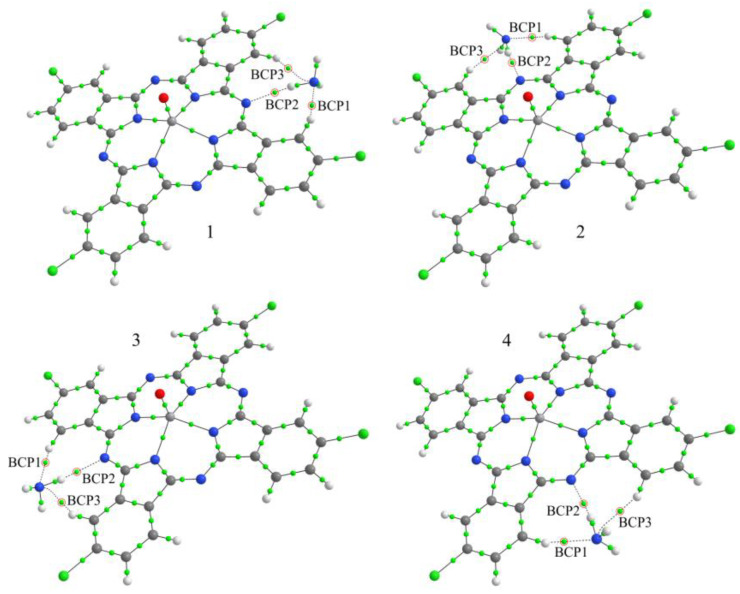
Structure of VOPcCl_4_-p/NH_3_-*x* (where *x* = 1–4) aggregates and bond critical points (BCPs, small green balls) in them. BCPs characterizing the interaction between the ammonia molecule and phthalocyanine atoms are shown in red circles.

**Figure 3:**
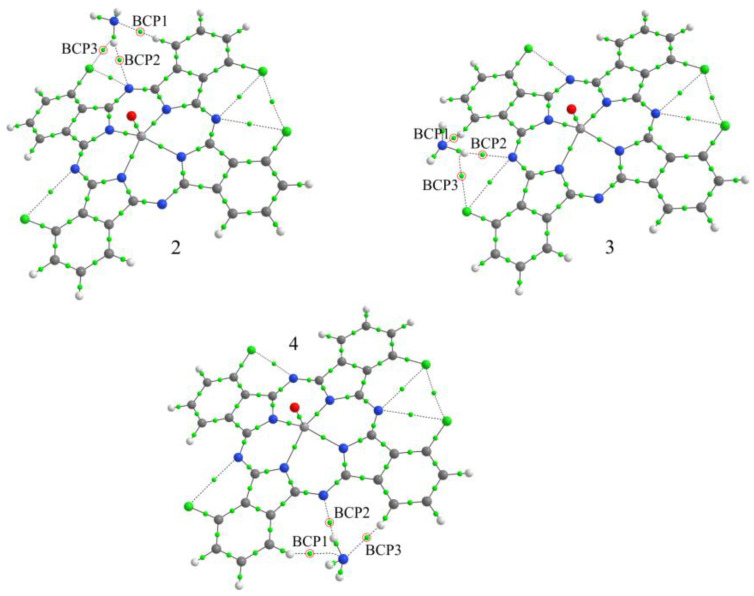
Structure of VOPcCl_4_-np/NH_3_-*y* (where *y* = 2–4) aggregates and bond critical points (BCPs, small green balls) in them. BCPs characterizing the interaction between the ammonia molecule and phthalocyanine atoms are shown in red circles.

The quantum-chemical approaches used here for the calculations of the geometric and electronic structures and total energies of VOPcCl_4_-p/NH_3_-*x* and VOPcCl_4_-np/NH_3_-*y* aggregates, where *x* = 1–4 and *y* = 2–4, have already been described in our previous work when performing a similar study of the interaction of ammonia molecule with fluorosubstituted phthalocyanines VOPcF_4_-p and VOPcF_4_-np [32]. The values of *x* and *y* specify the position of the NH_3_ molecule relative to the macrocycle (Figure 2 and Figure 3). From this point of view, the VOPcCl_4_-np/NH_3_-1 aggregate was not considered because it was shown [32] that such an arrangement of the molecule over the phthalocyanine plane cannot be realized due to the small distance between two adjacent macrocycles in the stack of molecules.

## 3. Results and Discussion

### 3.1. Crystal Structure of VOPcCl_4_-p and VOPcCl_4_-np

**Figure 4:**
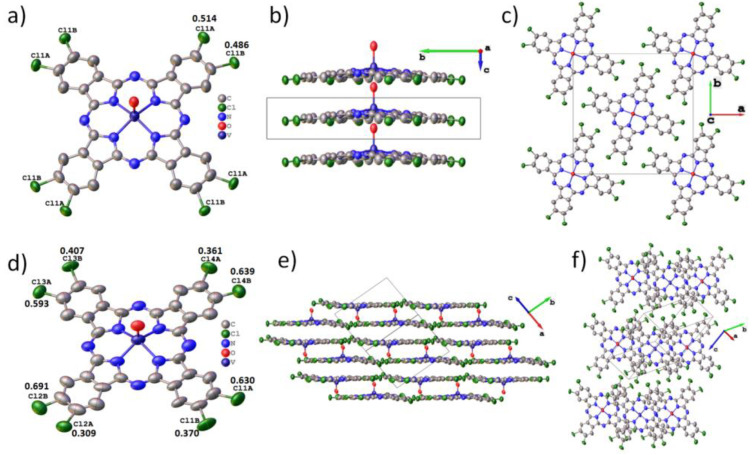
Molecular structure (**a**) and packing diagrams (**b**,**c**) for tetragonal polymorph and molecular structure (**d**) and packing diagrams (**e**,**f**) for triclinic polymorph of VOPcCl_4_-p.

Establishing the structure of single crystals is a necessary step before studying the structural features of thin films. Single crystals of vanadyl phthalocyanines were grown by sublimation in vacuum. Two types of single crystals were isolated from the VOPcCl_4_-p powder. Crystals in the form of very thin translucent greenish-blue ribbons were tetragonal, while needle-like crystals with a dark purple metallic sheen were in a triclinic phase. The molecular structure and packing diagrams for both VOPcCl_4_-p crystal phases are shown in Figure 4. Unit cell parameters and refinement details for VOPcCl_4_ crystal structures are summarized in Table 1. Due to the axial oxygen atom, the VOPcCl_4_-p molecule is not flat and has the shape of a “shuttlecock”, while the vanadium atom is displaced from the plane of the macrocycle by 0.399 Å, and the V = O bond is oriented perpendicular to the plane of the macrocycle (Figure 4a). Since VOPcCl_4_-p contains a mixture of regioisomers with peripheral chlorine atoms in different positions, each chlorine atom in the tetragonal crystal structure of VOPcCl_4_-p is disordered in two positions with a ratio of 0.514/0.486. VOPcCl_4_-p molecules in the tetragonal polymorph pack in vertical stacks along the c axis (Figure 4b,c) with the distance between adjacent molecules in a stack of 3.729 Å. The entire VOPcCl_4_-p molecule appears to be disordered by a mirror plane perpendicular to the tetragonality axis, so it seems that the VOPcCl_4_-p molecules are oriented “up” and “down” at the same time. This means that individual molecular stacks are randomly oriented either “up” or “down”, and there is no long-range symmetry in their ordering. This style of packing and disordering is not unique and was previously observed in crystals of halogenated lead phthalocyanines PbPcF_4_-p [38] and PbPcCl_4_-p [39].

Figure 4d shows a VOPcCl_4_-p molecule in triclinic polymorph. While the phthalocyanine core retains the same geometry as in the tetragonal polymorph, there is a clear difference in the distribution of peripheral chlorine atoms in alternative positions. While in the tetragonal polymorph, the distribution is close to 0.5/0.5, in the triclinic polymorph, this ratio is 0.691/0.309, which affects the molecular packaging. In the triclinic polymorph, the VOPcCl_4_-p molecules are assembled into chains, with the molecules alternating between an “up” and “down” orientation (Figure 4f). It is worth noting that the sites of chlorine atoms with low occupancy values (<0.5) are those between neighboring molecules in the chain, which means that close Cl…Cl contacts (<2.4 Å) between molecules in the same chain are most likely never present in a real crystal. Thus, the actual closest Cl…Cl contact between molecules in adjacent molecular chains in the triclinic VOPcCl_4_-p polymorph is 3.019 Å, which is still significantly shorter than in the tetragonal polymorph (3.364 Å). The distances between adjacent molecules in the chains are 3.217 Å and 3.299 Å. The individual molecular chains are arranged in the form of 2D layers that are packed parallel to the crystallographic plane (−222) (Figure 4e). The distance between molecules in adjacent layers is 3.095 Å, and the angle between the molecular layers and the plane (−222) is ~0.82°.

**Figure 5:**
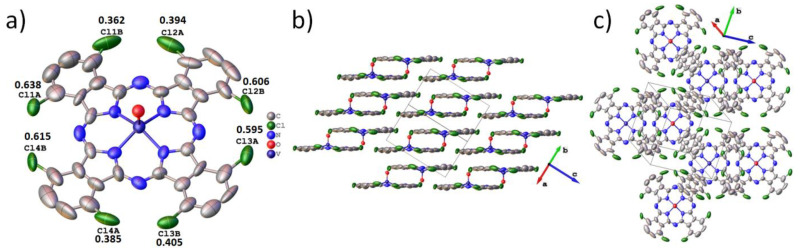
Molecular structure (**a**) and packing diagrams (**b**,**c**) for VOPcCl_4_-np.

VOPcCl_4_-np crystallizes in the space group P-1 with Z = 2 and Z’ = 1 and one symmetrically independent molecule in the unit cell (Table 1). The VOPcCl_4_-np molecular structure is shown in Figure 5a. The vanadium atom is displaced from the macrocycle plane by 0.553 Å, and the angle between the V=O bond and the normal to the macrocycle plane is 0.51°. Four chlorine atoms are disordered in two positions each. Their occupancy ratio is similar to the triclinic VOPcCl_4_-p polymorph and significantly deviates from the ratio 0.5/0.5.

VOPcCl_4_-np molecules are packed into two-dimensional layers parallel to the plane (2−22) (Figure 5b). The distance between molecules in one layer is 3.298 Å, and between adjacent layers, 3.239 Å. The angle between the molecular layers and the plane (2−22) is 0.15°. However, unlike VOPcCl_4_-p, VOPcCl_4_-np molecules do not form separate chains within one molecular layer but are arranged in such a way that each molecule faces three other molecules (Figure 5c).

### 3.2. VOPcCl_4_-p and VOPcCl_4_-np Thin Films and Powder XRD Study

**Figure 6:**
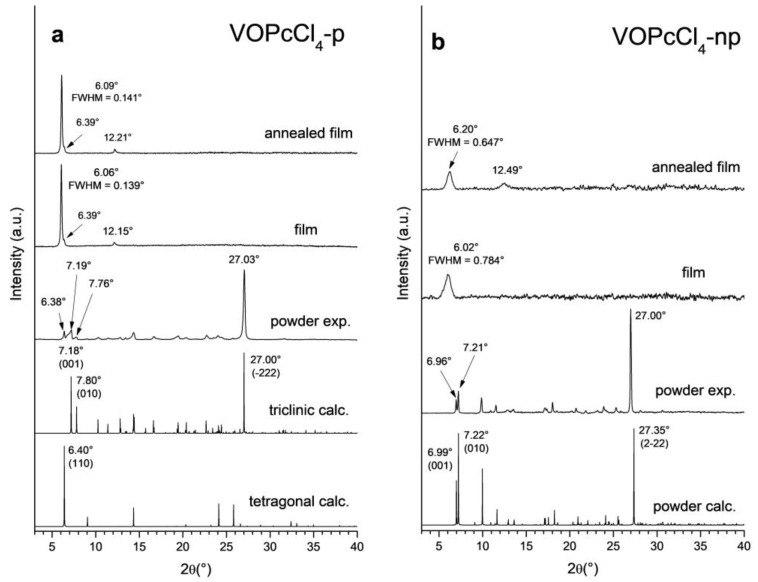
XRD patterns of bulk powders and thin films of VOPcCl_4_-p (**a**) and VOPcCl_4_-np (**b**).

Experimental XRD patterns of VOPcCl_4_-p and VOPcCl_4_-np powders and thin films (before and after annealing) are shown in Figure 6 in comparison with the powder patterns calculated based on the abovementioned single-crystal data. The first three peaks on the XRD pattern of the VOPcCl_4_-p powder (6.38°, 7.19°, and 7.76° 2θ) correspond to the plane (110) of the tetragonal phase and the planes (001) and (010) of the triclinic phase, indicating that both polymorph phases are present in a significant amount in the powder. The strong peak at 27.03° dominates the entire diffraction pattern and belongs to the plane (−222) of the triclinic polymorph, which suggests that the VOPcCl_4_-p powder consists mainly of the triclinic phase.

The first two peaks at 6.96° and 7.21° 2θ on the XRD pattern of VOPcCl_4_-np powder correspond to the planes (001) and (010), respectively, while the strong peak at 27.00° 2θ corresponds to the plane (2−22). The difference between the calculated and observed position for the peak (2−22) is caused by the difference in sample temperature during the experiment (150 K for SC-XRD and room temperature for PXRD). In general, the experimental powder patterns coincide well with the calculated ones for both VOPcCl_4_-p and VOPcCl_4_-np.

XRD patterns of both VOPcCl_4_-p and VOPcCl_4_-np films contain peaks that do not match the single-crystal data. The diffractogram of the VOPcCl_4_-p film is characterized by one strong diffraction peak at 6.06° 2θ and a peak at 12.15° 2θ with a weaker intensity, and the diffractogram of the VOPcCl_4_-np film contains a diffraction peak at 6.02° 2θ, which belongs to unknown crystal phases. The absence of other strong diffraction peaks indicates that the films have a preferred orientation. The difference in the phase composition of films from single crystals is often observed for phthalocyanines due to the tendency to form different polymorphic modifications depending on temperature [40]. To check the effect of the film annealing on their structure, the films were annealed at 200 °C for 2 h in air. Annealing resulted in a small shift in peak positions toward larger angles but did not change the overall appearance of the diffraction pattern, which can be caused by a release of mechanical stress and a decrease in the number of crystal lattice defects in thin films during annealing.

The optical absorption spectra of VOPcCl_4_-p and VOPcCl_4_-np films were also recorded. The spectra of VOPcCl_4_-p and VOPcCl_4_-np films are compared with the spectra of their solutions in dimethylformamide in Supplementary Figure S1. The spectra of VOPcCl_4_-p and VOPcCl_4_-np solutions are typical for the phthalocyanines, which are present in monomeric form in solutions [41], and have the Q bands with the maxima at 680 and 689 nm, respectively. The spectra of the films become broader because of intermolecular interactions in solids. Similar to the spectra of unsubstituted VOPc [42] and VOPcF_4_ [43] films, the maxima of Q bands shift to the higher wavelengths, i.e., to 778 and 795 nm of VOPcCl_4_-p and VOPcCl_4_-np films, respectively. This view of spectra is a characteristic of J-aggregate formation [44]. Films’ annealing at 200 °C for 2 h does not lead to noticeable changes in the intensity and position of the peaks.

VOPcCl_4_-p and VOPcCl_4_-np films were also characterized by Raman spectroscopy to demonstrate that their chemical composition coincides with that of the powders. Most of the peak positions in the spectra of the films coincide well with those in the spectra of powders (Supplementary Figure S2). The relative intensities of several peaks are different because of the preferred orientation in thin films, which is especially noticeable in the spectra of VOPcCl_4_-p samples.

**Figure 7:**
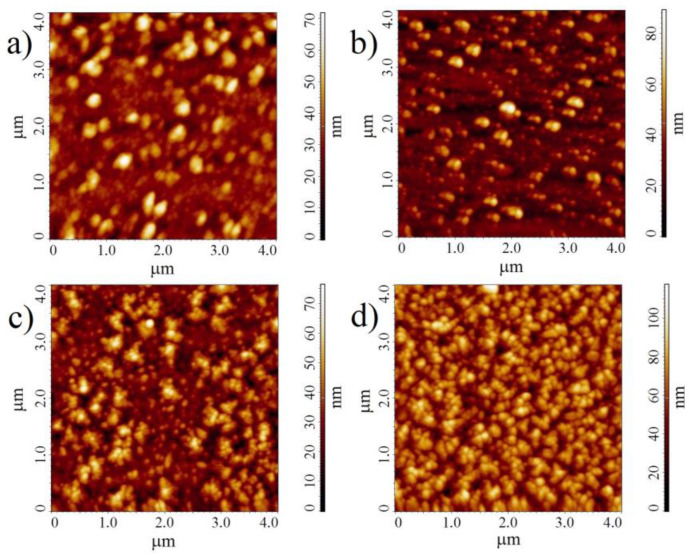
AFM images of VOPcCl_4_-p (**a**) and VOPcCl_4_-np (**c**) before annealing; and VOPcCl_4_-p (**b**) and VOPcCl_4_-np (**d**) after annealing.

The morphology of VOPcCl_4_-p and VOPcCl_4_-np films was studied by AFM. The microscopy images of the films before and after annealing (200 °C, 2 h) are shown in Figure 7.

The images show that both films are formed by spherical crystallites ranging in size from 50 to 200 nm. The root mean square (RMS) roughness value is 10.4 and 9.9 nm for the VOPcCl_4_-p and VOPcCl_4_-np films, respectively. After annealing in air, the crystallites increase slightly in size and become more clearly defined and more uniform in size. At the same time, the value of the RMS roughness of the VOPcCl_4_-p film decreases to 8.9 nm after annealing, but for VOPcCl_4_-np, it increases to 13.0 nm. The films were also characterized by the SEM method (Supplementary Figure S3). The surface of the films in the SEM images looks rough, with indistinctly pronounced rounded grains that become slightly larger after the heat treatment of the films.

### 3.3. Sensor Properties of VOPcCl_4_-p and VOPcCl_4_-np Films

#### 3.3.1. Comparison of the Sensor Response of VOPcCl_4_-p and VOPcCl_4_-np Films to Ammonia

**Figure 8:**
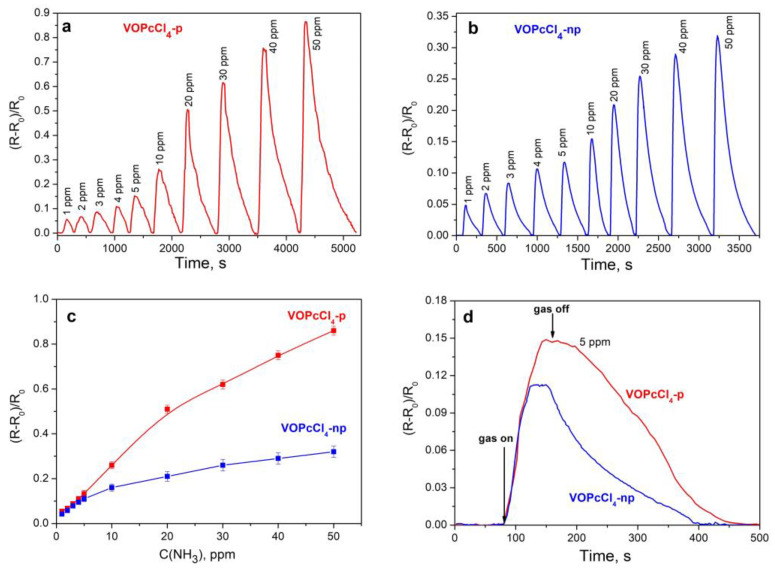
Curves of changes in the sensor response of VOPcCl_4_-p (**a**) and VOPcCl_4_-np (**b**) films when different concentrations of NH_3_ (1–50 ppm) are introduced in the flow cell (obtained in dynamic mode). Dependence of the sensor response of VOPcCl_4_-p (**a**) and VOPcCl_4_-np (**b**) films on NH_3_ concentration (**c**). Sensor response of VOPcCl_4_-p and VOPcCl_4_-np films to 5 ppm of NH_3_ (**d**), measured in the static mode. The measurements were carried out at room temperature (22 ± 2 °C).

The concentration of NH_3_ varied in the range from 1 to 50 ppm. The curves of changes in the sensor response, when different concentrations of NH_3_ were introduced, are shown in Figure 8a,b. The measurements of the sensor response were conducted in dynamic mode. In this mode, NH_3_ was injected at a constant airflow rate, and its exposure time was 15 s. The resistance of the films of both phthalocyanines increased after the introduction of ammonia into the cell, which was usually observed for metal phthalocyanine films exhibiting p-type semiconductor properties, for example, MPc and MPcF_4_ films [31]. The sensor response to NH_3_ was completely reversible at room temperature in the investigated concentration range. The dependence of the sensor response of VOPcCl_4_-p (a) and VOPcCl_4_-p (b) films on NH_3_ concentration is shown in Figure 8c. The change in the position of the chlorine substituents in the benzene rings strongly affects the value of the sensor response. For example, the value of the sensor response of a VOPcCl_4_-p film at 10 ppm is 1.6 times higher than that of a VOPcCl_4_-np film. A similar trend was previously found for chlorosubstituted zinc phthalocyanines [30]. The dependence of the response on the concentration is linear in the range from 1 to 10 ppm in the case of the VOPcCl_4_-p film and from 1 to 5 ppm in the case of the VOPcCl_4_-np film, thus making it possible to construct calibration curves in these concentration ranges and to calculate the limit of detection (LOD) according to the formula 3σ/m (where σ is the standard deviation of the sensor response to 1 ppm of ammonia, while m is the slope of the calibration plot). The calibration curves with the parameters used for the LOD calculation are presented in Supplementary Figure S4. The LODs were estimated to be 0.7 and 0.9 ppm for VOPcCl_4_-p and VOPcCl_4_-np films, respectively.

To determine the response and recovery times of the sensing layer, the static mode was used. The average response/recovery times measured at 5 ppm NH_3_ were 60/300 and 40/270 s for VOPcCl_4_-p and VOPcCl_4_-np films, respectively. The films of both phthalocyanines demonstrate good repeatability of the sensor response. For example, the value of the sensor response to 1 ppm of NH_3_ is 0.055 ± 0.003 when gas is repeatedly injected into the flow cell (Supplementary Figure S5).

#### 3.3.2. Study of the Nature of Interaction between NH_3_ and VOPcCl_4_ Molecules and Comparison of the Sensor Response of VOPcCl_4_ and VOPcF_4_ Films

Quantum-chemical calculations of the energy *E_b_* and the nature of NH_3_ binding in the corresponding VOPcCl_4_-p/NH_3_-*x* and VOPcCl_4_-np/NH_3_-*y* aggregates were performed to explain the difference in the sensor response of thin films of chlorosubstituted vanadyl phthalocyanines toward ammonia. In this case, the interaction of a gas molecule with macrocycles through their side atoms was considered (Figure 2 and Figure 3), since it was previously shown that this type of interaction best described the reversible experimental sensor response of the films of phthalocyanines with other substituents and metals [21,23]. It was shown as a result of the calculations that, depending on the position of NH_3_ relative to VOPcCl_4_-p, the *E_b_* values vary in the range from 0.171 eV to 0.180 eV (Table 2) and exceed the binding energies in the case of ammonia interaction with VOPcCl_4_-np (0.149–0.153 eV). The stronger binding between NH_3_ molecules and VOPcCl_4_-p bearing Cl substituents in peripheral positions correlates well with the higher sensor response of a VOPcCl_4_-p film to ammonia compared to VOPcCl_4_-np. A similar effect was observed earlier for tetrafluorosubstituted phthalocyanines [23].

The interaction of ammonia molecules with the side atoms of phthalocyanines occurs due to the formation of hydrogen bonds. This is evidenced by the values of the electron density *ρ*(**r**) and its Laplacian ∇^2^*ρ*(**r**) at the corresponding bond critical points (BCPs) (Figure 2 and Figure 3; Table 2). In particular, in the case of VOPcCl_4_-p/NH_3_, hydrogen bonds are formed between the hydrogen atoms of phthalocyanine and the ammonia nitrogen atom (BCP1 and BCP3) and between the bridging nitrogen atom of the macrocycle and one of the hydrogen atoms of the NH_3_ molecule (BCP2). The corresponding values of *ρ*(**r**) and ∇^2^*ρ*(**r**) are in the ranges of 0.091–0.147 *e*/Å^3^ and 0.974–1.559 *e*/Å^5^. Similar results were also obtained in the case of aggregates with chlorine atoms in nonperipheral positions of phthalocyanine macroring.

The difference is that in the VOPcCl_4_-np/NH_3_-2 and VOPcCl_4_-np/NH_3_-3 compounds, one of the hydrogen bonds and the corresponding BCP3 occur between one of the H atoms of NH_3_ and the Cl atom. In this case, the values of *ρ*(**r**) and ∇^2^*ρ*(**r**) at all considered BCPs are lower and equal to 0.060–0.105 *e*/Å^3^ and 0.793–1.566 *e*/Å^5^, respectively. Thus, the stronger NH_3_ molecule binding in VOPcCl_4_-p/NH_3_ aggregates compared to VOPcCl_4_-np/NH_3_, in particular, is due to higher electron density values in the corresponding BCPs. The conclusion that ammonia interacts with the side atoms of VOPcCl_4_ molecules by forming hydrogen bonds is because the values of *ρ*(**r**) and ∇^2^*ρ*(**r**) are in the ranges of 0.013–0.236 *e*/Å^3^ and 0.578–3.350 *e*/Å^5^, respectively (Table 2) [45].

**Figure 9:**
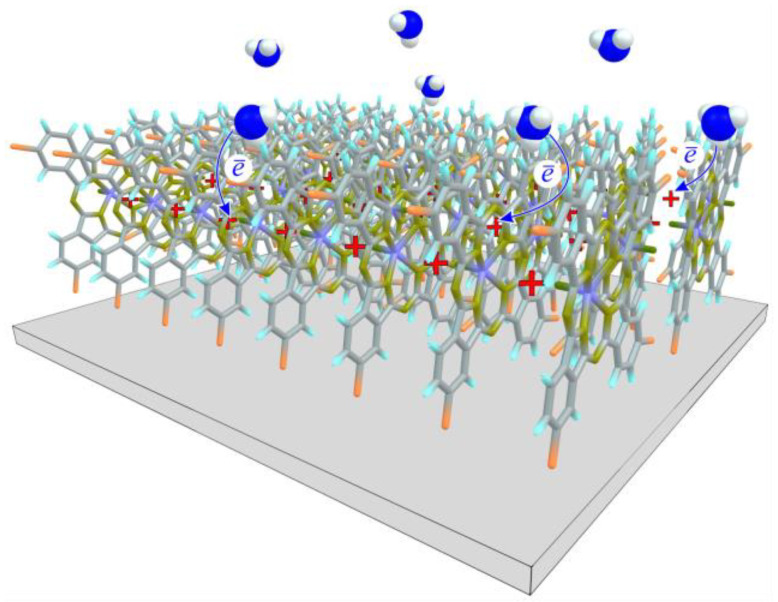
Scheme demonstrating the proposed mechanism of the sensor response. NH_3_ molecules donate electrons to macrocycles (blue arrows) due to interaction with their side atoms, leading to a decrease in the concentration of holes (red “plus” signs), i.e., charge carriers, and, consequently, to an increase in the resistance of phthalocyanine films.

The increase in resistance during the adsorption of NH_3_ molecules, which are electron donors, is typical for p-type semiconductors [46], which include thin films of tetrahalogensubstituted phthalocyanines. Therefore, the mechanism of sensor response can be represented using the scheme shown in Figure 9. Ammonia molecules donate electrons to VOPcCl_4_ macrocycles due to interaction with their side atoms, thus leading to a decrease in the concentration of holes, i.e., charge carriers, and, consequently, to an increase in the resistance of phthalocyanine films. The stronger the interaction or the higher the gas concentration, the greater the charge transfer and the higher the sensor response.

It should also be noted that the *E_b_* value of the NH_3_ molecule with VOPcCl_4_-p is slightly larger than with VOPcF_4_-p (0.170–0.174 eV), which was estimated earlier using the same computational approaches. This is consistent with a stronger sensor response of VOPcCl_4_ films compared to VOPcF_4_ ones. For example, the sensor response ((R − R_o_)/R_o_) of the VOPcCl_4_-p film was 0.15 vs. 0.14 (at 5 ppm NH_3_) in the case of a VOPcF_4_-p film [32]. This difference was even more noticeable at the higher ammonia concentrations (Supplementary Figure S6).

#### 3.3.3. Characteristics of the Sensor Based on VOPcCl_4_-p Films

**Figure 10:**
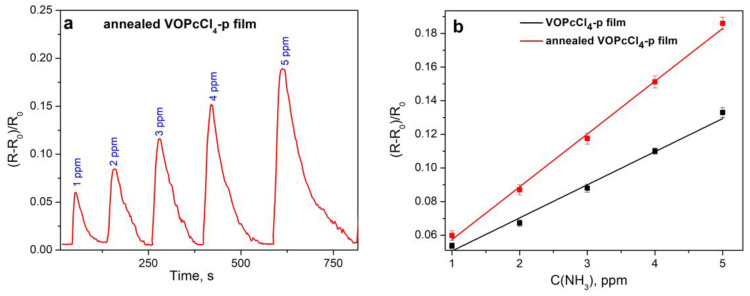
(**a**) Dynamical curve of the sensor response of the annealed VOPcCl_4_-p film to ammonia (1–5 ppm). (**b**) Sensor response of VOPcCl_4_-p film to ammonia (1–5 ppm) before (black line) and after (red line) annealing in air (200 °C, 2 h). The measurements were carried out at room temperature (22 ± 2 °C).

More detailed sensor characteristics were obtained for VOPcCl_4_-p films, which possess higher sensitivity to ammonia than VOPcCl_4_-np. First, the effect of annealing the films on the value of their sensor response to ammonia was investigated, since it was shown above that the annealing led to a change in the films’ morphology (Figure 7). The dynamical curve of the sensor response of the annealed VOPcCl_4_-p film is shown in Figure 10a. Figure 10b compares the dependencies of the sensor response of VOPcCl_4_-p films before and after annealing on the NH_3_ concentration ranging from 1 to 5 ppm. The annealing of VOPcCl_4_-p leads to an increase in the sensor response and the slope of the linear dependence of the sensor response on the concentration of NH_3_. As a result, the LOD was reduced to 0.3 ppm. The effect may be associated with an increase in the crystallinity of the films. It is known that the conductivity process in polycrystalline films of metal phthalocyanines is greatly influenced by the grain structure and size, as well as intergranular barriers. In several studies, it has been demonstrated that the higher the crystallinity of the film, the grain size, and the packing density of the crystallites, the greater the mobility of the charge carriers [47,48,49]. The electrical properties of polycrystalline films are determined by the capture of charge carriers in areas localized at the grain boundaries, resulting in a decrease in the free charge carriers involved in conduction and a significant decrease in their mobility due to scattering by potential barriers arising at the boundaries. The larger the average grain sizes in a polycrystalline film, the smaller this barrier. An increase in the charge carrier mobility in the VOPcCl_4_-p film may contribute to a stronger change in the resistance of the film when interacting with NH_3_. At the same time, the change in the sensor response of the VOPcCl_4_-np film after annealing is less pronounced than in the case of the VOPcCl_4_-p film. The recovery time for both vanadyl phthalocyanine films decreased after annealing. The recovery times for annealed VOPcCl_4_-p and VOPcCl_4_-np films (5 ppm) were 200 and 150 s, respectively.

**Figure 11:**
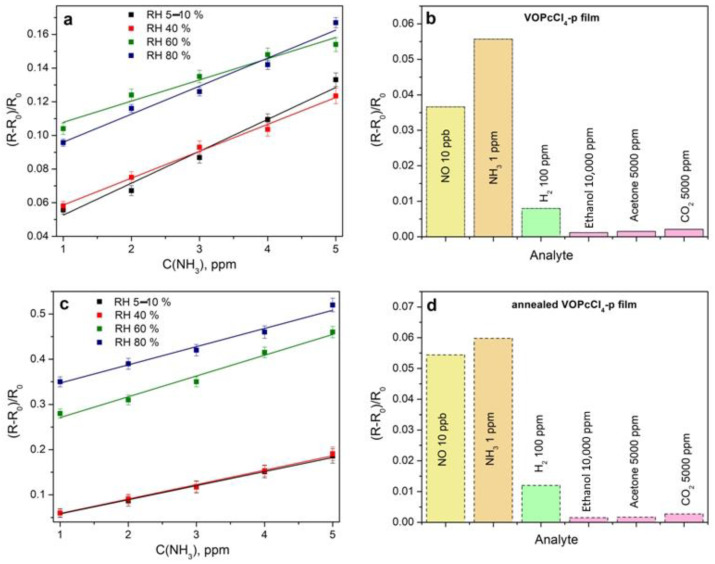
Sensor response of as-deposited and annealed VOPcCl_4_-p films to ammonia (1–5 ppm) at different levels of relative humidity (**a**,**c**) and to various gaseous analytes and volatile organic vapors (**b**,**d**). The measurements were carried out at room temperature (22 ± 2 °C).

The sensor response of as-deposited and annealed VOPcCl_4_-p films to ammonia (1–5 ppm) was tested at different levels of relative humidity (RH) (Figure 11a,c). With an increase in RH from 5 to 40%, the sensor response of both VOPcCl_4_-p films practically does not change; however, a further increase in RH to 60% leads to the growth of the sensor response by about 1.6 times. Similar to ammonia, the water molecule is also an electron donor; therefore, the simultaneous exposure of the film by the mixture of NH_3_ and H_2_O leads to an increase in its sensor response.

As already noted in the introduction, an increased level of ammonia in the exhaled air may be a sign of renal pathologies. The study of exhaled air for elevated levels of ammonia can be a convenient tool for the primary diagnosis of kidney diseases. To test the possibility of using phthalocyanine films in exhaled air, the measurements of their sensor response to some other gases (NO, CO_2_, and hydrogen) and volatile organic compounds (VOCs) that may be present in exhaled air, as well as to NH_3_ in a mixture of gases similar in composition to exhaled air, were carried out. The diagrams exhibiting the sensor response of as-deposited and annealed VOPcCl_4_-p films to these analytes in comparison with ammonia are shown in Figure 11b,d. Similarly to fluorosubstituted metal phthalocyanines [31], the VOPcCl_4_-p films exhibit a significantly higher response to NH_3_ than to the investigated VOCs and other gases, except for nitric oxide (Figure 11b,d). Note that the concentration of these gases is much higher than that of ammonia. In this case, NO is an interfering gas, and the detection of ammonia may be difficult, even if it is present in the gas mixture at the ppb level. This is especially important to take into account when studying biomarker gases in exhaled air. For example, in a healthy person, the NO concentration can be found in the concentration range of 10–50 ppb, while the ammonia concentration can rise from 0.5 ppm in a healthy person to around 2 ppm in the case of liver or kidney failure [50]. Therefore, the effect of the presence of nitric oxide on the sensor response to ammonia is the subject of further investigation.

**Figure 12:**
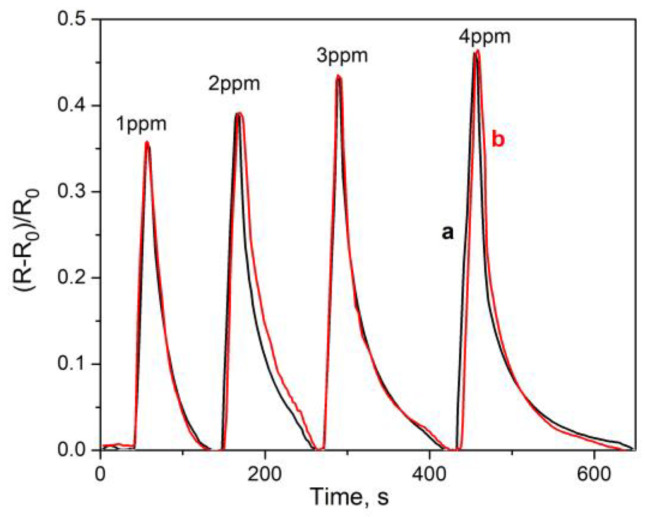
The sensor response of a VOPcCl_4_-p film to ammonia (1–4 ppm) in moist air (a) and in a mixture of gases similar in composition to exhaled air (b). RH of the moist air and the gas mixture was the same and was maintained at 80–85%. The measurements were carried out at room temperature (22 ± 2 °C).

Figure 12 shows the sensor response of an annealed VOPcCl_4_-p film to ammonia (1–4 ppm) in a mixture of gases simulating exhaled air in comparison with that measured in moist air. This mixture contained N_2_ (76%), O_2_ (16%), H_2_O (5%), and CO_2_ (3%). It should be noted that the relative humidity of the moist air and the gas mixture was the same and was maintained at 80–85%. It can be seen from Figure 11 that the sensor response to ammonia in this mixture is almost the same as in moist air. Thus, films based on chlorinated vanadyl phthalocyanines can potentially be used to detect the low ammonia concentration in human-exhaled air. Tests of real exhaled samples are beyond the scope of this work, as they require the involvement of other methods to study their composition.

To test the long-term stability of the sensor, the response of the annealed VOPcCl_4_-p films was measured after 1, 2, 7, and 30 days (see Supplementary Figure S7). It was shown that the films demonstrate good stability, and the change in their sensor response after a fairly long time was within the experimental error.

The sensor characteristics of as-deposited and annealed VOPcCl_4_-p films were compared with other sensors based on metal phthalocyanines and relative compounds studied in recent works (Table 3). It can be seen that both the as-deposited and annealed VOPcCl_4_-p films can compete with sensors based on films of other metal phthalocyanines, and the sensitivity of the annealed films even exceeds the value of the sensor response of the films presented in Table 3. Only halogenated cobalt and zinc phthalocyanine films studied in our previous works [30,32,51] had a lower detection limit than the VOPcCl_4_-p films, but the recovery time of the VOPcCl_4_-p films is lower (150 s) and the value of the sensor response (52%) is higher compared to that of the CoPcF_4_-p films (215 s and 41%, respectively) and ZnPcF_4_-p (210 s and 16%, respectively). Thus, this study makes it possible to expand the range of metal phthalocyanines, which are promising materials for use as active layers of chemiresistive sensors to ammonia.

Our analysis of review articles devoted to the study of ammonia sensors showed that resistive sensors based on semiconductor oxides and their hybrid materials have a lower response (4–30 s) and relaxation times (10–90 s) compared to sensors based on fluorosubstituted phthalocyanines; however, their detection limits are usually worse and vary (i.e., 0.1–20 ppm) [57,58]. Sensors of ammonia based on OFET structures [57] with conductive polymers as sensing layers have low LODs (usually from tens to hundreds parts per billion) that are comparable with phthalocyanine films, but usually their response and recovery times are quite high and range from several minutes to tens of minutes.

As a rule, the active layers of sensors with better characteristics than those of phthalocyanine films are not single-component films but mixtures of complex organic semiconductors [59,60] or complex heterostructures, for example, oxides or conductive polymers modified with nanoparticles or carbon nanomaterials [61,62]. Compared with such heterostructures, the advantage of phthalocyanine films lies in the simplicity of the production method.

## 4. Conclusions

In this work, the films of tetrachlorosubstituted vanadyl phthalocyanine derivatives were studied as active layers for detecting low concentrations of NH_3_. Tetrachlorosubstituted vanadyl phthalocyanines with Cl substituents in the peripheral (VOPcCl_4_-p) and nonperipheral (VOPcCl_4_-np) positions of the phthalocyanine ring were investigated to reveal the effect of the position of substituents on the structure of their films prepared by a physical vapor deposition technique and their sensor response to ammonia. It was shown that the films of VOPcCl_4_-p exhibited a noticeably higher sensor response to both NH_3_ films when compared to VOPcCl_4_-np films. This was explained with the help of quantum chemical calculations by the stronger interaction of NH_3_ molecules with the side atoms of phthalocyanine having peripheral Cl substituents as compared to the phthalocyanines bearing the substituents in the nonperipheral position of the macrocycle. The limit of detection (LOD) of NH_3_ was 0.7 ppm. The postdeposition annealing of the films allowed us to increase the sensor response and reduce the detection limit to 0.3 ppm. The sensing layers demonstrated a reversible sensor response at room temperature, with fairly low response/recovery times of 50/300 s at 5 ppm NH_3_. The fact that NH_3_ can be detected in the presence of various interfering gases and vapors, as well as in a mixture of gases similar in composition to exhaled air, indicates that VOPcCl_4_ films may be good candidates for their use as active layers for detecting ammonia as a biomarker in exhaled air. However, a lot of effort is still needed to improve their selectivity, as well as the response time, which is higher than that of most sensors based on semiconductor oxides. Therefore, further efforts should be directed to testing phthalocyanine-based sensing layers and their arrays in multicomponent gas mixtures and to study hybrid materials of metal phthalocyanines with metal nanoparticles and/or carbon nanomaterials, which are known to contribute to improving sensor performance.

## Figures and Tables

**Table 1:** Unit cell parameters and refinement details for VOPcCl_4_ crystal structure.

Compound	VOPcCl_4_-p (Tetragonal)	VOPcCl_4_-p (Triclinic)	VOPcCl_4_-np
Empiric formula	C_32_H_12_Cl_4_N_8_OV	C_32_H_12_Cl_4_N_8_OV	C_32_H_12_Cl_4_N_8_OV
Formula weight	717.24	717.24	717.24
Temperature/K	150	150	150
Crystal system	Tetragonal	Triclinic	Triclinic
Space group	I4/m	P-1	P-1
a/Å	19.5370(10)	9.139(4)	8.9851(16)
b/Å	19.5370(10)	12.716(6)	12.505(2)
c/Å	3.7577(2)	14.291(6)	12.937(2)
α/°	90	114.719(10)	99.740(5)
β/°	90	106.252(11)	96.323(5)
γ/°	90	94.796(11)	95.171(6)
Volume/Å^3^	1434.29(17)	1409.5(11)	1415.1(4)
Z	2	2	2
ρ_calc_ g/cm^3^	1.661	1.690	1.683
μ/mm^−1^	0.764	0.777	0.774
F(000)	718.0	718.0	718.0
Crystal size/mm^3^	0.13 × 0.03 × 0.005	0.14 × 0.02 × 0.02	0.03 × 0.03 × 0.01
Radiation	MoK_α_ (λ = 0.71073)	MoK_α_ (λ = 0.71073)	MoK_α_ (λ = 0.71073)
2Θ range for data collection/°	4.17 to 51.29	4.78 to 46.874	4.59 to 46.536
Index ranges	−21 ≤ h ≤ 23, −23 ≤ k ≤ 23, −4 ≤ l ≤ 4	−10 ≤ h ≤ 10, −13 ≤ k ≤ 14, −15 ≤ l ≤ 15	−9 ≤ h ≤ 9, −13 ≤ k ≤ 13, −13 ≤ l ≤ 14
Reflections collected	7682	13,062	13,781
Independent reflections	791 [R_int_ = 0.0506, R_sigma_ = 0.0263]	4063 [R_int_ = 0.1507, R_sigma_ = 0.1820]	4054 [R_int_ = 0.1984, R_sigma_ = 0.2193]
Data/restraints/parameters	791/2/107	4063/0/455	4054/12/431
Goodness-of-fit on F^2^	1.055	0.968	0.954
Final R indexes [I ≥ 2σ (I)]	R_1_ = 0.0600, wR_2_ = 0.1659	R_1_ = 0.0881, wR_2_ = 0.2129	R_1_ = 0.0918, wR_2_ = 0.2038
Final R indexes [all data]	R_1_ = 0.0833, wR_2_ = 0.1868	R_1_ = 0.2140, wR_2_ = 0.2839	R_1_ = 0.2459, wR_2_ = 0.2858
Largest diff. peak/hole/e Å^−3^	0.76/−0.30	0.53/−0.41	1.16/−0.33
CCDC №	2267526	2267527	2267528

**Table 2:** The interaction parameters of the NH_3_ molecule with vanadyl phthalocyanines.

Compound	*E_b_*, eV	BCP	Atoms *	*ρ*(r), *e*/Å^3^	∇^2^*ρ*(r), *e*/Å^5^
VOPcCl_4_-p/NH_3_-1	0.174	1	H-N	0.147	1.559
		2	N-H	0.139	1.365
		3	H-N	0.094	1.011
VOPcCl_4_-p/NH_3_-2	0.180	1	H-N	0.137	1.489
		2	N-H	0.134	1.323
		3	H-N	0.094	1.015
VOPcCl_4_-p/NH_3_-3	0.180	1	H-N	0.137	1.491
		2	N-H	0.134	1.326
		3	H-N	0.094	1.016
VOPcCl_4_-p/NH_3_-4	0.171	1	H-N	0.139	1.511
		2	N-H	0.136	1.337
		3	H-N	0.091	0.974
VOPcCl_4_-np/NH_3_-2	0.149	1	H-N	0.105	1.259
		2	N-H	0.102	1.105
		3	Cl-H	0.060	0.789
VOPcCl_4_-np/NH_3_-3	0.153	1	H-N	0.105	1.261
		2	N-H	0.101	1.104
		3	Cl-H	0.060	0.793
VOPcCl_4_-np/NH_3_-4	0.153	1	H-N	0.101	1.090
		2	N-H	0.122	1.228
		3	H-N	0.143	1.566

* The first atom belongs to the phthalocyanine molecule, while the second atom belongs to the NH_3_ molecule.

**Table 3:** Sensor characteristics of as-deposited and annealed VOPcCl_4_-p films in comparison with other sensors based on metal phthalocyanines and relative compounds.

Layers	Sensor Response, % *	LOD, ppb	Linear Range, ppm	Response Time, s	Recovery Time, s	Refs.
VOPcF_4_-p	14 (5 ppm)	40	1–10	48 (at 5 ppm)	270 (at 5 ppm)	[32]
CoPcF_4_-p	41 (5 ppm)	10	1–10	55	215	[32]
ClAlPc	11.5 (1 ppm)	100	0.1–1	60 (fixed)	n/a	[52]
Cl_2_SiPc/LuPc_2_	13 (10 ppm)	100	10–90	<60 (at 90 ppm)	<420 (at 90 ppm)	[53]
TiOTPP **	25 ± 2 (1 ppm)	50	0–0.75	90	1200	[54]
Tetrakis(n-octylthio) phthalocyaninato copper(II)	~20 (10 ppm)	n/a	10–50	50 (at 30 ppm)	30 (at 30 ppm)	[55]
p-isopropylbenzene/LuPc_2_	~20 (10 ppm)	n/a	10–90	60 (fixed)	240 (fixed)	[56]
CoPcR_8_ ***	2.8 (5 ppm)	30	0.3–50	20 (at 5 ppm)	40 (at 5 ppm)	[51]
ZnPcF_4_-p	16 (5 ppm)	10	0.1–50	45 (at 1 ppm)	210 (at 1 ppm)	[30]
ZnPcCl_4_-p	18 (5 ppm)	10	0.1–50	45 (1 ppm)	260 (1 ppm)	
VOPcCl_4_-p	15 (5 ppm)	70	1–10	60 (at 5 ppm)	200 (at 5 ppm)	This work
Annealed VOPcCl_4_-p	52 (5 ppm)	30	1–10	50 (at 5 ppm)	150 (at 5 ppm)	This work

* The sensor response is defined as (R − R_o_)R_o_⋅100% and provided for those concentrations that are indicated in the corresponding reference; ** TPP is tetraphenylporphyrin; *** R is 5-(trifluoromethyl)-2-mercaptopyridine.

## Data Availability

The data presented in this study are available upon request from the corresponding author. VOPcCl_4_-p and VOPcCl_4_-np structural data can be obtained from https://www.ccdc.cam.ac.uk/structures/, accessed on 13 September 2023 (numbers 2267526, 2267527, and 2267528).

## Supplementary Materials

The following supporting information can be downloaded at https://www.mdpi.com/article/10.3390/mi14091773/s1, Figure S1. Optical absorption spectra of the solutions of VOPcCl4-p and VOPcCl4-np in dimethylformamide (black lines) and their films before (blue lines) and after (red lines) heat treatment; Figure S2. Raman spectra of VOPcCl4-p and VOPcCl4-np films (blue lines) and powders (black lines); Figure S3. SEM images of VOPcCl4-p and VOPcCl4-np (black lines) and their films before (a,c) and after (b,d) heat treatment; Figure S4. Dependencies of the sensor response and fitting parameters used for the calculation of LODs of VOPcCl4-p and VOPcCl4-np films; Figure S5. Repeatability of the sensor response of a VOPcCl4-p film, measured at 1 ppm of NH3; Figure S6. Comparison of the dependence of the sensor response on ammonia concentration for VOPcCl4-p and VOPcF4-p films; Figure S7. The sensor response of an annealed VOPcCl4-p film to 1 ppm of NH3, measured in 1, 2, 7, and 30 days.
