# Supplementary material for: Thin Films of Chlorinated Vanadyl Phthalocyanines as Active Layers of Chemiresistive Sensors for the Detection of Ammonia
Source: Micromachines (Basel). 2023 Sep 15;14(9):1773. doi: 10.3390/mi14091773 (PMC10534441; doi:10.3390/mi14091773)
Supplement: Supplementary file 1 [file micromachines-14-01773-s001.zip › micromachines-2549705-supplementary.pdf]

Supporting Information

Thin films of chlorinated vanadyl phthalocyanines as active layers of chemiresistive sensors for the detection of ammonia

Darya Klyamer[1], Alexandr Sukhikh[1], Dmitry Bonegardt[1], Pavel Krasnov[2], Pavel Popovetskiy[1], Tamara Basova[1]*

[1] Nikolaev Institute of Inorganic Chemistry SB RAS, 3 Lavrentiev Pr., 630090 Novosibirsk, Russia; niic@niic.nsc.ru
[2] International Research Center of Spectroscopy and Quantum Chemistry, Siberian Federal University, 26 Kirensky st., 660074 Krasnoyarsk, Russia
* Correspondence: basova@niic.nsc.ru;

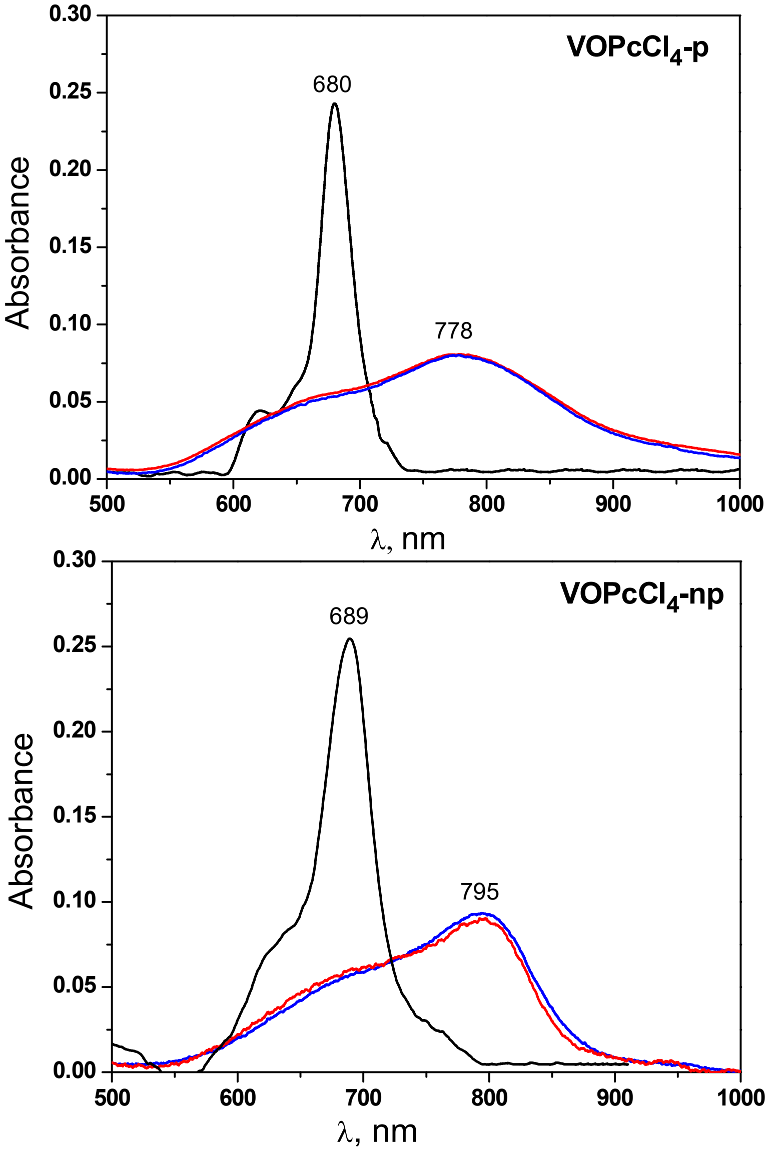

Figure S1. Optical absorption spectra of the solutions of $VOPcCl_4$-p and $VOPcCl_4$-np in dimethylformamide (black lines) and their films before (blue lines) and after (red lines) heat treatment.

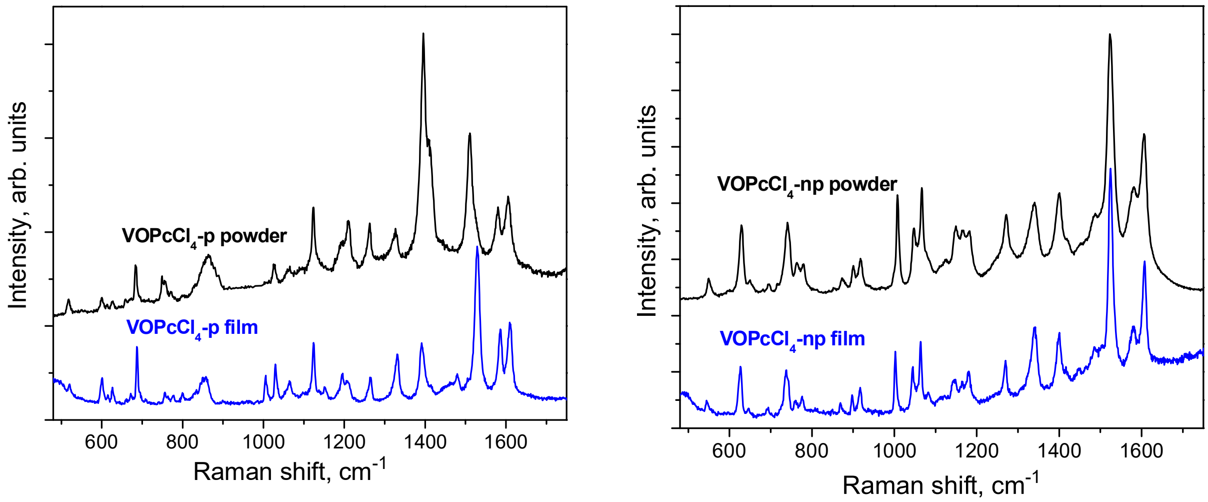

Figure S2. Raman spectra of VOPcCl4-p and VOPcCl4-np films (blue lines) and powders (black lines).

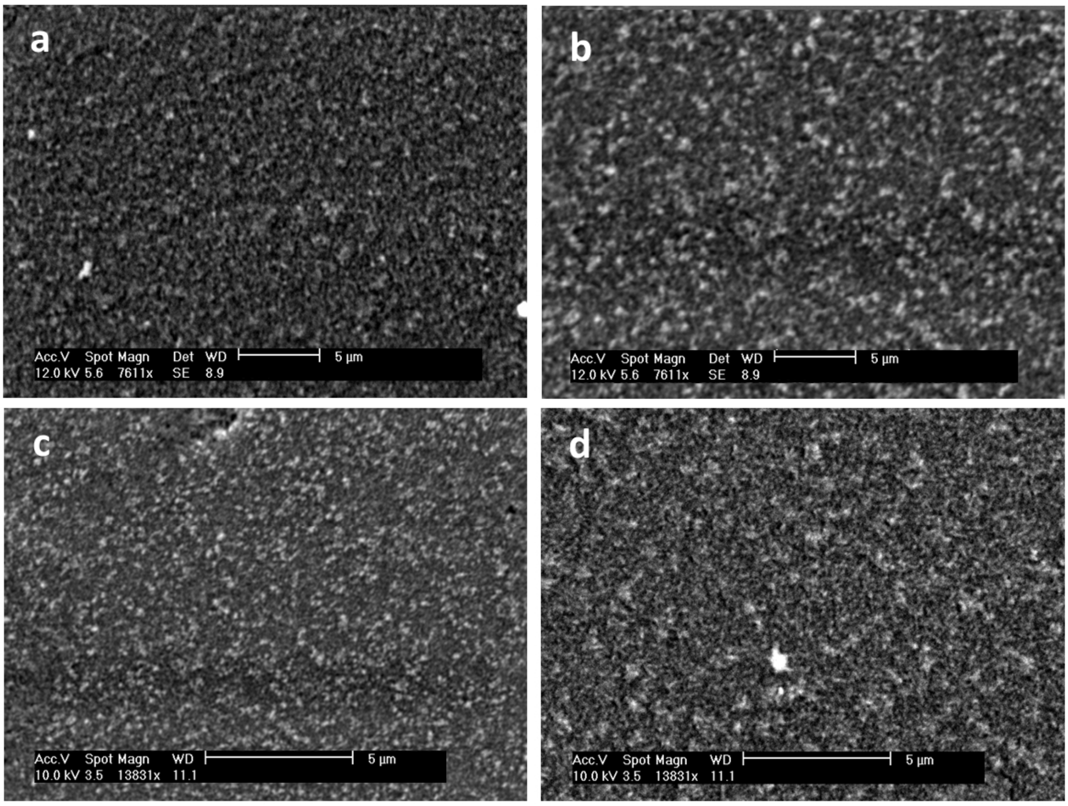

Figure S3. SEM images of VOPcCl4-p and VOPcCl4-np (black lines) and their films before (a, c) and after (b, d) heat treatment.

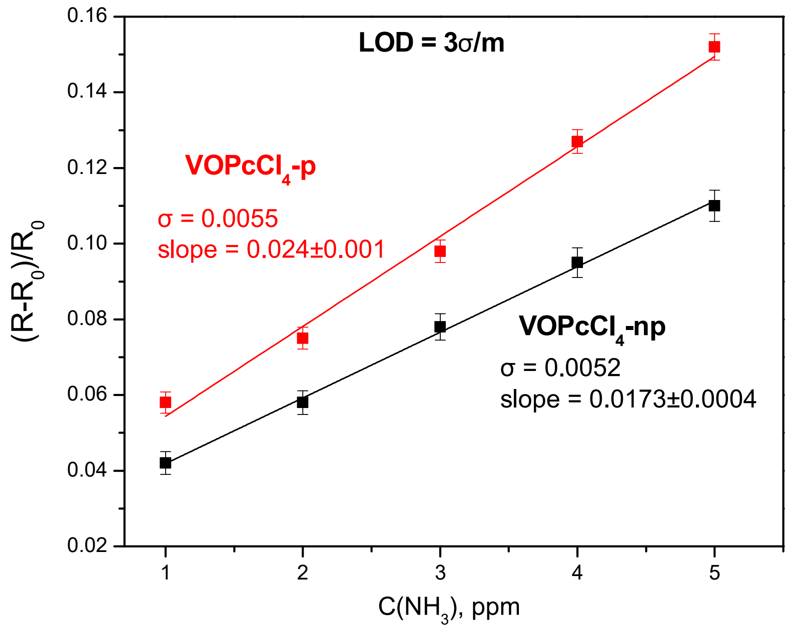

Figure S4. Dependencies of the sensor response and fitting parameters used for the calculation of LODs of $VOPcCl_4$-p and $VOPcCl_4$-np films.

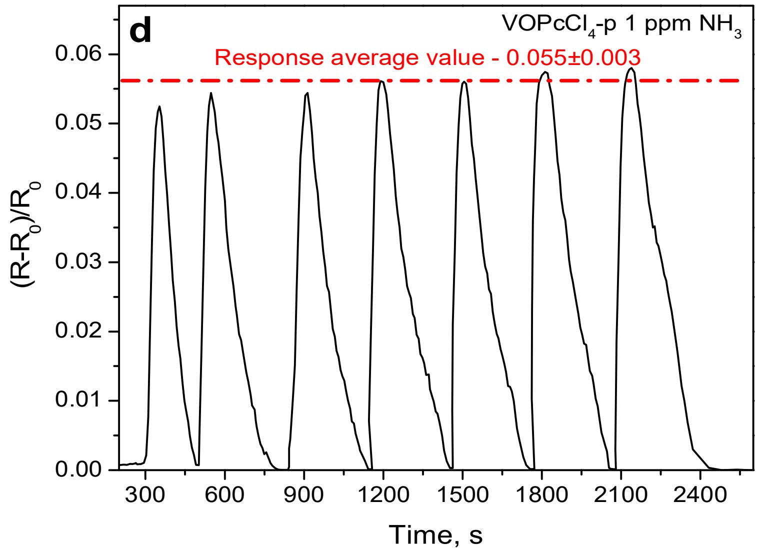

Figure S5. Repeatability of the sensor response of a $VOPcCl_4$-p film, measured at 1 ppm of NH_3.

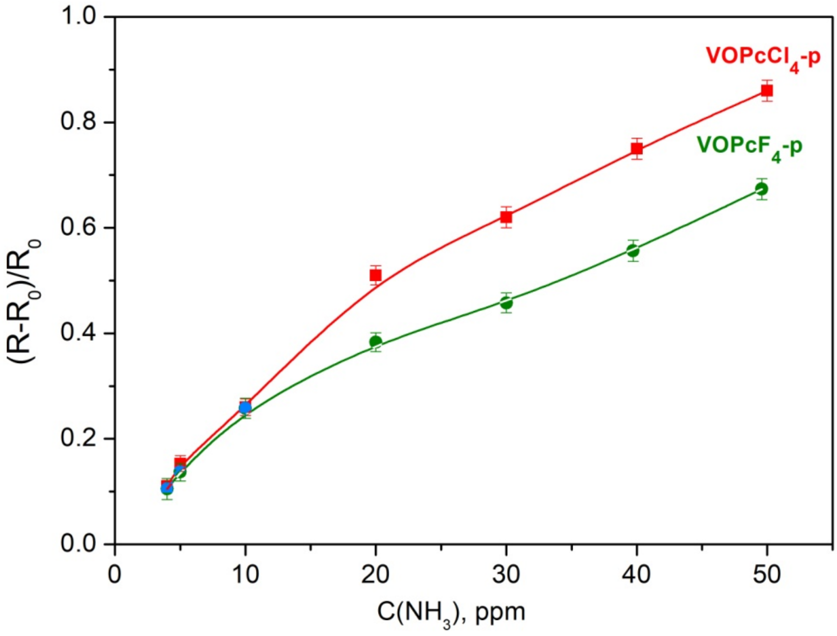

Figure S6. Comparison of the dependence of the sensor response on ammonia concentration for VOPcCl$_4$-p and VOPcF$_4$-p films.

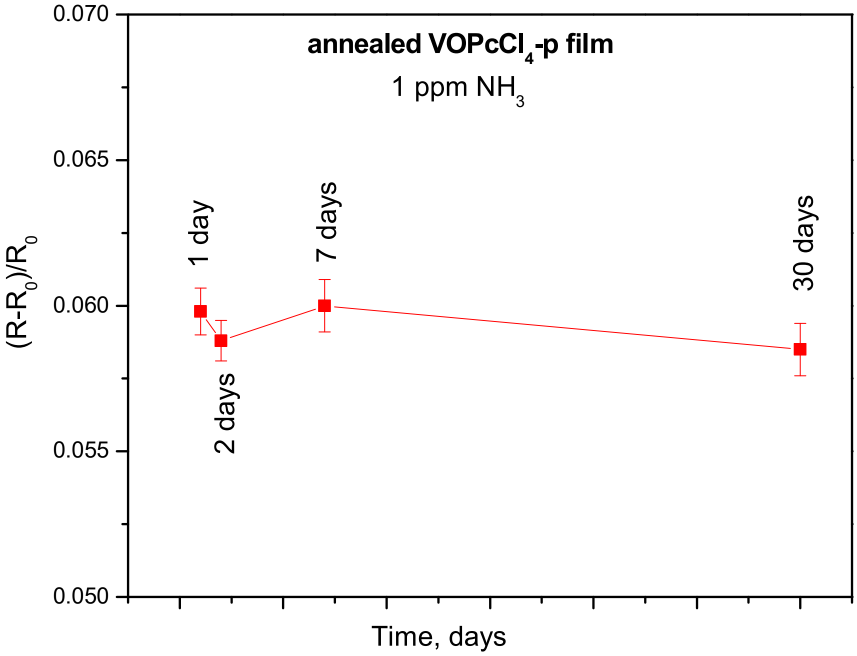

Figure S7. The sensor response of an annealed VOPcCl$_4$-p film to 1 ppm of NH_3, measured in 1, 2, 7 and 30 days.
